# Identification of Dipeptidyl-Peptidase (DPP)5 and DPP7 in *Porphyromonas endodontalis*, Distinct from Those in *Porphyromonas gingivalis*

**DOI:** 10.1371/journal.pone.0114221

**Published:** 2014-12-10

**Authors:** Haruka Nishimata, Yuko Ohara-Nemoto, Tomomi T. Baba, Tomonori Hoshino, Taku Fujiwara, Yu Shimoyama, Shigenobu Kimura, Takayuki K. Nemoto

**Affiliations:** 1 Department of Oral Molecular Biology, Course of Medical and Dental Sciences, Nagasaki University Graduate School of Biomedical Sciences, Nagasaki, Japan; 2 Department of Pediatric Dentistry, Course of Medical and Dental Sciences, Nagasaki University Graduate School of Biomedical Sciences, Nagasaki, Japan; 3 Division of Molecular Microbiology, Iwate Medical University, Yahaba-cho, Iwate, Japan; United States Army Medical Research Institute of Infectious Diseases, United States of America

## Abstract

Dipeptidyl peptidases (DPPs) that liberate dipeptides from the N-terminal end of oligopeptides are crucial for the growth of *Porphyromonas* species, anaerobic asaccharolytic gram negative rods that utilize amino acids as energy sources. *Porphyromonas endodontalis* is a causative agent of periapical lesions with acute symptoms and Asp/Glu-specific DPP11 has been solely characterized in this organism. In this study, we identified and characterized two *P. endodontalis* DPPs, DPP5 and DPP7. Cell-associated DPP activity toward Lys-Ala-4-methylcoumaryl-7-amide (MCA) was prominent in *P. endodontalis* ATCC 35406 as compared with the *Porphyromonas gingivalis* strains ATCC 33277, 16-1, HW24D1, ATCC 49417, W83, W50, and HNA99. The level of hydrolysis of Leu-Asp-MCA by DPP11, Gly-Pro-MCA by DPP4, and Met-Leu-MCA was also higher than in the *P. gingivalis* strains. MER236725 and MER278904 are *P. endodontalis* proteins belong to the S9- and S46-family peptidases, respectively. Recombinant MER236725 exhibited enzymatic properties including substrate specificity, and salt- and pH-dependence similar to *P. gingivalis* DPP5 belonging to the S9 family. However, the *k*_cat_/*K*_m_ figure (194 µM^−1^·sec^−1^) for the most potent substrate (Lys-Ala-MCA) was 18.4-fold higher as compared to the *P. gingivalis* entity (10.5 µM^−1^·sec^−1^). In addition, *P. endodontalis* DPP5 mRNA and protein contents were increased several fold as compared with those in *P. gingivalis*. Recombinant MER278904 preferentially hydrolyzed Met-Leu-MCA and exhibited a substrate specificity similar to *P. gingivalis* DPP7 belonging to the S46 family. In accord with the deduced molecular mass of 818 amino acids, a 105-kDa band was immunologically detected, indicating that *P. endodontalis* DPP7 is an exceptionally large molecule in the DPP7/DPP11/S46 peptidase family. The enhancement of four DPP activities was conclusively demonstrated in *P. endodontalis*, and remarkable Lys-Ala-MCA-hydrolysis was achieved by qualitative and quantitative potentiation of the DPP5 molecule.

## Introduction

Previous studies have reported a high prevalence of *Porphyromonas endodontalis*, a gram-negative black-pigmented anaerobe, in infected root canals with acute symptoms [1]–[3] as well as specimens obtained from orofacial odontogenic infections [4], though most of the pathogenic factors of this organism have yet to be elucidated. *Porphyromonas* species are asaccharolytic, and utilize amino acids as energy and carbon sources [5]. Amino acids are mainly incorporated as di- and tri-peptides through membrane transporters [6]–[8]. Since *P. endodontalis* seems to have no potent endopeptidase activity [2], [9], dipeptidyl-peptidases (DPPs) that liberate dipeptides from the N-terminus of oligopeptides can be crucial for the entire metabolism of this bacterium. In addition, previous studies have reported that the end metabolites of amino acids, e.g., propionate and butyrate, in *Porphyromonas* species facilitate dental plaque development [10], and exert cytotoxicity to pulp [11] and inflamed gingival fibroblasts [12]. Therefore, DPPs are also considered to be key enzymes involved in pathogenicity related to these bacteria.

It is important to address the extracellular oligopeptides-degradation system as a mechanism to elucidate how asaccharolytic organisms survive under an oligotrophic environment, such as in a root canal. Along this line, a comparison of peptidases of *P. endodontalis* with those of *P. gingivalis*, a causative agent of aggressive forms of adult periodontitis, is of interest. A number of studies have investigated *P. gingivalis* peptidases and shown that *P. gingivalis* prominently produces trypsin-like cysteine endopeptidases, i.e., Arg- and Lys-specific gingipains [13]–[15], in contrast to the absence of such peptidases in *P. endodontalis*.

All four DPPs known to be expressed in *P. gingivalis* belong to either S9- or S46-family peptidase [16]–[21]. Although both family peptidases are serine peptidases and form an active triad composed of His Asp and Ser, the topology and positions of the three residues are completely distinct from each other, *i.e.*, Ser^542^, Asp^627^, and His^659^ in S9-family *P. gingivalis* DPP5 and His^89^, Asp^225^ [21], and Ser^648^ in S46-family *P. gingivalis* DPP7 [19].

DPPs generally attack oligopeptides without N-terminal modification and sequentially liberate dipeptides from the N-terminus. The penultimate P1-position residue from the N-terminus is critical for the recognition by DPPs, although the N-terminal P2-position residue additionally affects the activity [20]. Among them, DPP4 solely liberates the glycylprolyl dipeptide among DPPs [16], [17]. Furthermore, hydrophobic amino acid-specific DPP7 of the S46 family has also been characterized [19], [20]. We recently identified the third and fourth *P. gingivalis* DPPs, DPP11 and DPP5 [18], [21]. The former is a novel S46-family enzyme that specifically cleaves a peptide bond of penultimate Asp and Glu [21], while the latter belongs to the S9 family and is specific for the P1-position Ala and hydrophobic amino acids [18]. Although DPP5 was initially discovered in fungi, such as *Aspergillus fumigatus* [22] and *Microsporum canis* [23], the finding of *P. gingivalis* DPP5 expanded its distribution from fungi to eubacteria and archea, as well as higher plants and animals [18]. Since these four DPPs exhibit distinguished P1 and P2 preferences for each other, they are able to cover most combinations of the dipeptide repertoire [18], [20].

In *P. endodontalis*, the DPP11 gene was cloned, and the biochemical and enzymatic properties were well characterized [21]. In addition, the hydrolytic activity of Gly-Pro-*p*-nitroanilide [24] and an 88-kDa angiotensin degrading endopeptidase [25] have been reported, though these precise characteristics and amino acid sequences have to be elucidated. Based on the genomic shotgun assembly sequence of *P. endodontalis* ATCC 35406 [26], the MEROPS peptidase database now lists 57 putative and known peptidases [27]. In accordance with phylogenetic analysis indicating that every bacterial species of the phylum *Bacteroidetes* possesses each gene of DPP7 and DPP11 [20], *P. endodontalis* possesses the gene of DPP11 and another encoding MER278904, possibly DPP7, though it is only designated as an unassigned peptidase of the S46 family. This ambiguity is mainly due to an eccentrically large open reading frame of MER278904 encoding 818 amino acid residues as compared to the average 717±24 residues (mean±S.D., n = 264) of the S46 family members. In addition, four S9 peptidase family genes encoding MER192286 (DPP4), MER236725, MER237803, and MER326507 are enrolled in the database.

In the present study, cell-associated DPP activities in *P. endodontalis* were determined, and compared with those of laboratory and clinical strains of *P. gingivalis*. In agreement with a previous study [9], *P. endodontalis* predominantly hydrolyzed Lys-Ala-MCA among the dipeptidyl substrates tested and the activity was markedly higher than that of *P. gingivalis*. We also identified and characterized *P. endodontalis* DPP5 and DPP7 by expressing the unassigned genes, and demonstrated that hydrolyzing of Lys-Ala-MCA is mediated by DPP5 (PeDPP5/MER236725) and *P. endodontalis* DPP7 (PeDPP7/MER278904) is expressed as an exceptionally large molecule. Biochemical properties of both DPPs were highly distinct from their respective *P. gingivalis* entities.

## Materials and Methods

### Bacterial strains and culture conditions

*P. endodontalis* ATCC 35406 and *P. gingivalis* ATCC 33277 and ATCC 49417 were obtained from American Type Culture Collection. *P. gingivalis* strains 16-1, HW24D1, HG1690, W83, W50, and HNA99 were preserved in our laboratories. *P. gingivalis* KDP136 is a disruptant of all three-gingipain genes in ATCC 33277 [28]. Bacterial cells were grown in an anaerobic condition (80% N_2_, 10% CO_2_, 10% H_2_) at 36°C using anaerobic bacteria culture medium (Eiken Chemical, Tokyo, Japan) until the early stationary phase. Then, aliquots were inoculated onto enriched Tryptic Soy agar (Nissui, Tokyo, Japan) plates supplemented with 0.5% brain-heart infusion broth (BBL, Sparks, MD), 5 µg/mL hemin (Nacalai Tesque, Kyoto, Japan), and 0.5 µg/mL menadione (Sigma-Aldrich, St. Louis, MO) covered with a sterilized dialysis membrane (6-kDa cut-off) for easy harvest of the bacterial fraction free from broth components [29], [30]. After two days, bacterial cells were suspended in ice-cold phosphate-buffered saline, pH 7.4 (PBS), and centrifuged at 10,000×*g* for 10 minutes at 4°C. Cell pellets were washed once with PBS, and then the resultant pellet was re-suspended in PBS adjusted to an absorbance at 600 nm of 2.0 and used for the experiments.

### Expression and purification of recombinant DPPs

*P. endodontalis* genomic DNA was used as a template for PCR cloning, as previously reported [21]. A DNA fragment encoding Lys^2^-Lys^691^ of putative PeDPP5 (MER236725) was amplified with a pair of primers (5′-GACTAAGGATCCAAAAGAACTATCCTCAGCCTC-3′ and 5′GCATCAGGATCCTTTTTTGAGCCAACGGTCCAG-3′) carrying *Bam*HI sites (underlined). A DNA fragment encoding the N-terminal part (Leu^3^-Lys^710^) of putative PeDPP7 (MER278904 encoding Met^1^-Lys^818^) was amplified by PCR using a set of primers (5′-AAATTCCCATGGAGCTAAAAAGAATCT-3′ and 5′-CGCGTTCCATGGCCTTTACGAGGTTGAGTT-3′) carrying *Bgl*II sites (underlined). After digestion with either *Bam*HI or *Bgl*II, the fragments were cloned into the *Bam*HI site of pQE60 (Qiagen, Valencia, CA). Expression plasmids for *P. gingivalis* DPP5 (PgDPP5), DPP7 (PgDPP7), and DPP11 (PgDPP11), as well as that of *P. endodontalis* DPP11 (PeDPP11) were previously reported [18], [20], [21]. DPPs were expressed in *E. coli* XL-1 Blue cultured in Luria-Bertani broth supplemented with 75 µg/mL of ampicillin by induction with 0.2 mM isopropyl-thiogalactopyranoside at 30°C for 4 hours. Recombinant proteins were purified by Talon affinity chromatography as previously reported [21].

### Measurement of peptidase activity

Peptidase activity was measured as previously reported [18], [21]. Briefly, the reaction was started by addition of a cellular fraction (5 µL) or DPPs (50 ng) to 200 µL of a reaction mixture composed of 50 mM sodium phosphate (pH 7.0), 5 mM EDTA, and 20 µM dipeptidyl MCA. Leu-Asp-MCA was synthesized by Thermo Fisher Scientific (Ulm, Germany), and other substrates were purchased from Peptide Inst. (Osaka, Japan) and Bachem (Dubendorf, Switzerland). NaCl (0.1 M) was added for measurement of the activity of DPP5. After 30 minutes at 37°C, fluorescence intensity was measured with excitation at 380 nm and emission at 460 nm. In some experiments, pH values varied from 5 to 8.5 and NaCl concentrations from 0 to 1.6 M. To determine the enzymatic parameters, recombinant proteins (5 ng) were incubated with various concentrations of dipeptidyl MCA (0.5–160 µM) and the parameters were calculated by fitting a nonlinear regression curve to the Michaelis-Menten equation using the GraphPad Prism software program (San Diego, CA).

### Immunoblotting of DPP5 and DPP7

Rabbit anti-PgDPP7 antiserum was prepared as previously reported [21]. Recombinant DPPs or cell lysate whole proteins were separated on SDS-PAGE, then subjected to Coomassie brilliant blue (CBB) staining. Alternatively, separated proteins were transferred to polyvinylidene difluoride membranes (Merck Millipore, Darmstadt, Germany), and subjected to immunoblotting with anti-PgDPP5 [16] or anti-PgDPP7 serum. Protein bands were incubated with alkaline phosphatase-conjugated anti-rabbit IgG (Promega, Madison, WI), and visualized with 5-bromo-4-chloro-3-indolyl phosphate and nitro blue tetrazolium. To detect PeDPP7, blotted membranes were incubated with anti-PgDPP7 serum, then with a peroxidase-conjugated anti-rabbit IgG antibody (Promega), and the color was finally developed with 4-chloro-1-naphthol to avoid development of a nonspecific 100-kDa band.

### Quantification of mRNA levels

Total RNA was isolated using Isogen reagent (Nippon Gene, Tokyo, Japan). DNA contaminating the preparation was digested with DNase I (Nippon Gene) for 15 minutes at 37°C. cDNA was prepared from DNA-free RNA (1 µg) with superscript III reverse transcriptase (Life Technology, Grand Island, NY) in a reaction mixture (20 µL) containing 1.3 mM dNTPs and 50 ng of random primers (Takara Bio, Shiga, Japan). The expression levels of DPP5 mRNA were quantitated by qPCR as previously reported [31]. The following primers were used: for PeDPP5, 5′-CCAACCGACCTCTACCGCAT-3′ and 5′-CGCAACGAATGTCTCCCAGC-3′; for PgDPP5, 5′-CATTCTTGCTTCACAGGCTATGG-3′ and 5′-TGCATTCTGCGGTTGGAGT-3′; for *P. endodontalis* 16S ribosome RNA, 5′-AGGAGACGAGGTATGCGGAA-3′ and 5′-TCGTGCTTCAGTGTCAGACG-3′; and for *P. gingivalis* 16S ribosome RNA, 5′-GAGGGACAAAGGGCAGCTAC-3′ and 5′-TGCGCGATTACTAGCGAATCC-3′. The reaction was started at 94°C for 10 minutes followed by 40 cycles consisting of denaturation at 94°C for 30 seconds, annealing at 60°C for 30 seconds, and extension at 72°C for 1 minute. pGEM T-easy plasmids (Promega) carrying 110- and 114-bp parts of *P. endodontalis and P. gingivalis* 16S ribosome RNA, respectively, were constructed by TA cloning with the respective PCR fragments. These plasmids and the DPP5 expression plasmids were used to standardize and calculate the copy numbers of PeDPP5 and PgDPP5 mRNA.

### Inhibition enzyme-linked immunosorbent assay (ELISA)

An inhibition ELISA was performed to determine the concentration of DPP5 in whole lysates. Briefly, standard curves were obtained with recombinant PeDPP5 or PgDPP5(1 ng–3 µg) in 50 µg of total proteins adjusted with bovine serum albumin in PBS. The reactivity of anti-PgDPP5 antibody against PeDPP5 was 12.1% of that against PgDPP5. *P. endodontalis* or *P. gingivalis* cell suspensions corresponding to 50 µg of proteins was separately incubated in 96-well plates. After blockage with blocking buffer [0.17 M H_3_BO_3_, pH 8.5, 0.12 M NaCl, 0.05% (v/v) Tween20, 1 mM EDTA, 0.25% (w/v) bovine serum albumin, 0.05% (w/v) NaN_3_], the wells were processed with anti-PgDPP5 (1,000-fold dilution) and peroxidase-conjugated anti-rabbit IgG (2 µg/mL) antibodies, each for 30 minutes at 37°C. Absorbance at 655 nm was measured after color development at 37°C for 30 minutes with SureBlue TMB Microwell Peroxidase Substrate (1-Component) (Kirkegaard and Perry Lab., Gaitherburg, MD).

## Results

### Peptidase activities and dipeptide production profiles in *P. endodontalis* and *P. gingivalis*

**Figure 1:**
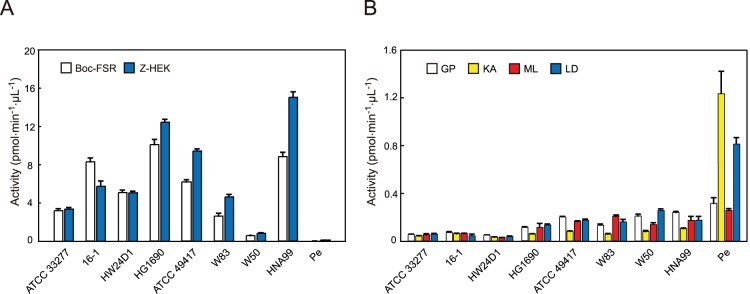
Cell-associated peptidase activity profiles of *P. endodontalis* and eight strains of *P. gingivalis*. (A) Aliquots (5 µL) of bacterial cell suspensions (*A_600_* = 2.0) of *P. gingivalis* strains and *P. endodontalis* ATCC 35406 were incubated in a reaction mixture containing 20 µM Boc-Phe-Ser-Arg- or Z-His-Glu-Lys-MCA. (B) DPP activities toward Gly-Pro-, Lys-Ala-, Met-Leu-, and Leu-Asp-MCA were measured. Activity values are shown as the mean±S.D. (n = 3).

Hydrolysis of benzyloxycarbonyl (Z)-His-Glu-Lys-MCA that was characteristic of Lys-gingipain (Kgp) and *t*-butyloxycarbonyl (Boc)-Phe-Ser-Arg-MCA of Arg-gingipain (Rgp) was commonly demonstrated in the eight strains of *P. gingivalis* (ATCC 33277, 16-1, HW24D1, HG1690, ATCC 49417, W83, W50, HNA99), though some variations of these activities were observed. Strains HNA99, HG1690, ATCC 49417, and 16-1 showed relatively high activities for both gingipains, while strain W50 showed the lowest level of hydrolysis (Fig. 1). The absence of gingipain-like activities was obvious in *P. endodontalis* ATCC 35406, in agreement with previous reports [2], [9]. The DPP activities of these *Porphyromonas* strains were examined using four different fluorescent synthetic dipeptidyl substrates, Gly-Pro-, Lys-Ala-, Met-Leu-, and Leu-Asp-MCA, which are specific or preferential substrates of DPP4, DPP5, DPP7, and DPP11, respectively [18]. They were hydrolyzed within a few variations of efficiency by the *P. gingivalis* strains (Fig. 1). Interestingly, the hydrolyzing activities for Leu-Asp-, Gly-Pro-, and Met-Leu-MCA were higher in *P. endodontalis* than those respective ones for all of the *P. gingivalis* strains, with the hydrolysis of Lys-Ala-MCA in particular the most prominent in *P. endodontalis*.

Although both DPP4 and DPP7 can hydrolyze Lys-Ala-MCA [20], [32], we recently reported that DPP5 dominantly hydrolyzes this substrate in *P. gingivalis* [18]. A comparison of amino acid sequences of listed S9 peptidase family genes of *P. endodontalis* strongly suggested that MER236725 is an orthologue of PgDPP5 (61.4% identity, PGN_0756), and the other two (MER237803, MER326507) are prolyl tripeptidyl-peptidase A [33] (48.1%, PGN_1149), and Ala-DPP [34] (43.8%, PGN_1694), respectively. Therefore, it is now commonly expected that the most predominant Lys-Ala-MCA hydrolyzing activity in *P. endodontalis* is also mediated by DPP5, thus the putative DPP5 gene of the microorganism was searched for, as shown in the following section.

### Identification and characterization of putative PeDPP5 (MER236725)

**Figure 2:**
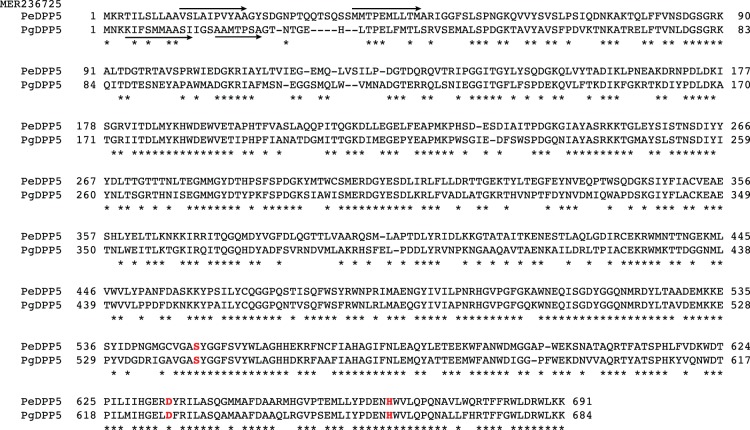
Alignment of amino acid sequences of *P. endodontalis* MER236725 and *P. gingivalis* DPP5. The amino acid sequences of putative *P. endodontalis* DPP5 (PeDPP5, MER236725) and *P. gingivalis* DPP5 (PgDPP5, PGN_0756/MER034615) were aligned by Genetyx. Hyphens represent gaps introduced for maximal matching. Common amino acids are marked by asterisks. Three amino acids essential for serine proteases are written in red letters. Arrows indicate the N-terminal amino acid sequences determined with recombinant proteins.

**Figure 3:**
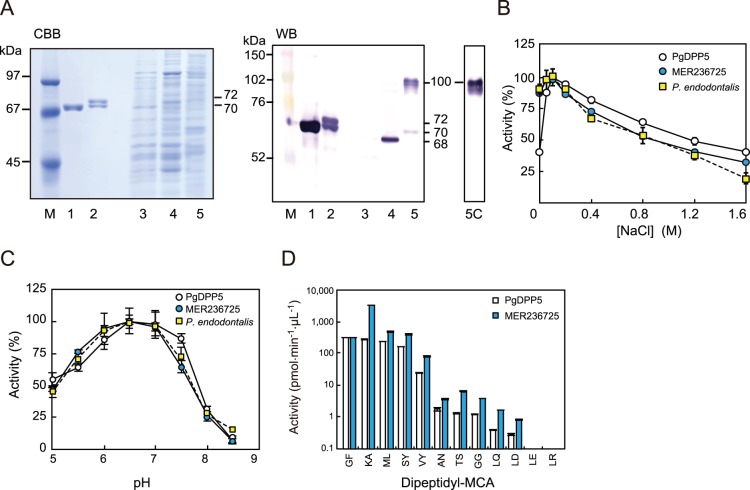
Biochemical analysis of putative PeDPP5 (MER236725). (A) Recombinant proteins (0.3 µg) of PgDPP5 (lane 1), putative PeDPP5 (lane 2), and aliquots (5 µL) of bacterial cell suspensions of *P. gingivalis* ATCC 33277 (lane 3), KDP136 (lane 4), and *P. endodontalis* (lanes 5 and 5C) were separated on SDS-PAGE gels, then stained with CB or subjected to immunoblotting (WB). The 100-kDa bands in lanes 5 and 5C were non-specifically observed with the alkaline phosphatase-conjugated second antibody. Lane M, molecular-weight markers. (B) NaCl-concentration dependence and (C) pH dependence of the hydrolyzing activity toward Lys-Ala-MCA of PgDPP5, putative PeDPP5 (MER236725), and *P. endodontalis* cells were determined. (D) The peptidase activities of recombinant PgDPP5 and PeDPP5 were determined using various dipeptidyl MCAs. Values are shown as the mean±S.D. (n = 3).

MER236725, referred to as putative *P. endodontalis* DPP5 (PeDPP5), shows the conserved catalytic triad of Ser^579^, Asp^634^, and His^666^ (Fig. 2). A full-length form of MER236725 expressed in *E. coli* migrated as 70- and 72-kDa doublets (Fig. 3). The N-terminal sequence of the 70-kDa molecule was M^37^MTPEMLLTM^45^ and that of the 72-kDa molecule was V^12^SLAIPVYAA^21^. Immunoblotting analysis demonstrated that MER236725 cross-reacted with the anti-PgDPP5 antibody. In bacterial whole cell lysates, expression of a 70-kDa entity was demonstrated in accord with the deduced molecular mass of 74,374, while *P. gingivalis* DPP5 was observed as a single 68-kDa species in KDP136. No band was shown in ATCC 33277. The enzymatic activity of MER236725 toward Lys-Ala-MCA was maximal in the presence of 0.1 M NaCl at pH 6.7 (Fig. 3B and C). These salt and pH dependencies were apparently similar to those of PgDPP5. Moreover, the substrate repertoire of MER236725 was comparable to that of PgDPP5, though the activity of MER236725 was much higher than that of PgDPP5 (Fig. 3D). Taken together, these findings identified MER236725 as PeDPP5. Furthermore, DPP5 actually accounted for most of the hydrolysis of Lys-Ala-MCA in *P. endodontalis*, since the pH and salt dependencies of the activity were indistinguishable between the bacterial cells and recombinant PeDPP5 (Fig. 3B, C).

Determination of the enzymatic parameters of PeDPP5 for four major substrates revealed that it possessed the smallest *K*_m_ and highest *k*_cat_ values for Lys-Ala-MCA, resulting in the maximal *k*_cat_/*K*_m_ value of 194 µM^−1^·sec^−1^ (Table 1) which was 18.5-fold higher than that of PgDPP5 (10.5 µM^−1^·sec^−1^). The superiority of PeDPP5 to PgDPP5 was also observed with Met-Leu- and Ser-Tyr-MCA, and the *k*_cat_/*K*_m_ values of Gly-Phe-MCA were comparable (Table 1, Fig. 3).

**Table 1:** Enzymatic parameters of PeDPP5 and PgDPP5.

Species	MCA substrate	*k*_cat_ (sec^−1^)	*K*_m_ (µM)	*k*_cat_/*K*_m_ (µM^−1^sec^−1^)
*P. endodontalis*	Lys-Ala-	21213±932	110±7	194
	Met-Leu-	5175±193	196±11	26.4
	Ser-Tyr-	3468±87	257±9	13.5
	Gly-Phe-	10378±1635	993±176	10.5
*P. gingivalis*	Lys-Ala-	1948±165	185±21	10.5
	Met-Leu-	5562±701	701±104	7.93
	Ser-Tyr-	1329±100	296±31	4.49
	Gly-Phe-	5638±403	396±51	13.0

Averages and standard errors were calculated from four independent measurements.

### mRNA and protein levels of DPP5

**Figure 4:**
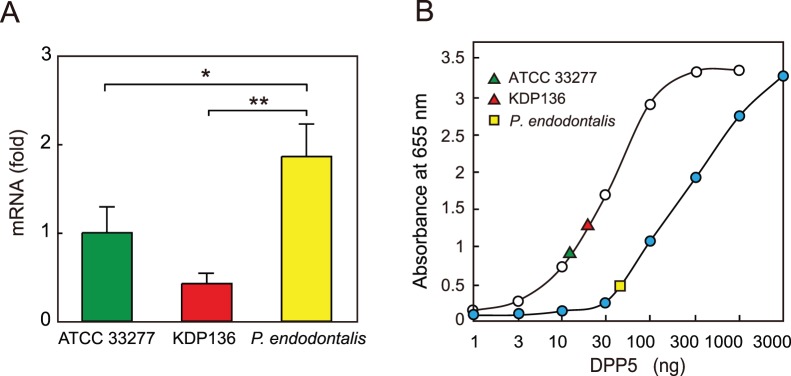
DPP5 mRNA and protein levels. (A) Relative amounts of DPP5 mRNA in *P. gingivalis* ATCC 33277, KDP136, and *P. endodontalis* were measured by qPCR. Values are shown as the mean±S.D. (n = 3). *p<0.05; **p<0.01. (B) Inhibition ELISA was performed with recombinant PgDPP5 (white circles) and PeDPP5 (blue circles), as described in Materials and Methods. The amounts of DPP5 in *P. gingivalis* ATCC 33277, KDP136, and *P. endodontalis* whole cell lysates were determined to be 12.0±3.0, 20.9±3.5, and 51.8±12.8 ng/µg total proteins, respectively (mean±S.D., n = 3).

We also quantitated DPP5 mRNA and protein levels in *P. endodontalis* and *P. gingivalis*. Quantitative RT-PCR revealed a significantly higher level of PeDPP5 mRNA (1.9- and 4.4-fold) as compared to those in *P. gingivalis* ATCC 33277 and KDP136, respectively (Fig. 4). An inhibition ELISA using anti-PgDPP5 antiserum also revealed that the DPP5 protein contents in *P. endodontalis* and *P. gingivalis* ATCC 33277 and KDP136 were 51.8, 12.0, and 20.9 ng/µg of total proteins, respectively, suggesting that the content was 2–5-fold higher in *P. endodontalis* than in *P. gingivalis*.

### Expression and characterization of putative PeDPP7 (MER278904)

**Figure 5:**
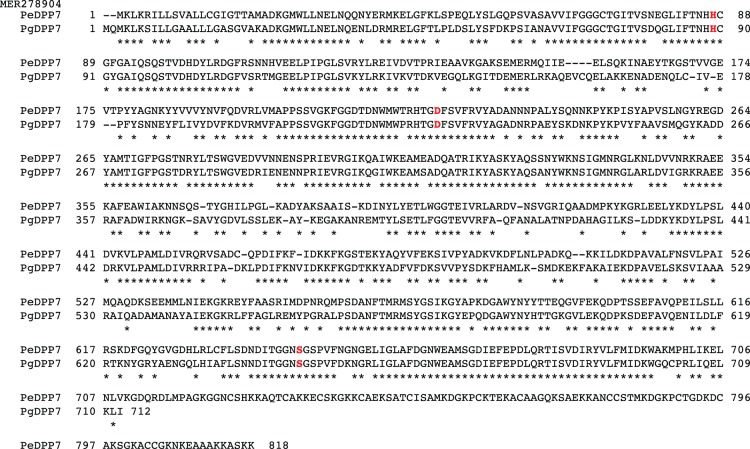
Alignment of amino acid sequences of *P. endodontalis* MER278904 and *P. gingivalis* DPP7. The amino acid sequences of putative *P. endodontalis* DPP7 (PeDPP7, MER278904) and *P. gingivalis* DPP7 (PgDPP7, PGN_1479/MER014366) were aligned by Genetyx. Hyphens represent gaps introduced for maximal matching. Common amino acids are marked by asterisks. Three amino acids essential for serine proteases are written in red letters.

Another unassigned S46-family peptidase of *P. endodontalis*, MER278904, consists of the N-terminal region (Met^1^-Val^709^), which showed an amino acid sequence similarity to the S46 family members as well as the C-terminal extra unrelated sequence (Lys^710^-Lys^818^) (Fig. 5). The N-terminal region showed the highest identity to PgDPP7 (65.3%), indicating that MER278904 represents PeDPP7, in which the catalytic triad composed of His^87^, Asp^223^, and Ser^645^ was conserved.

**Figure 6:**
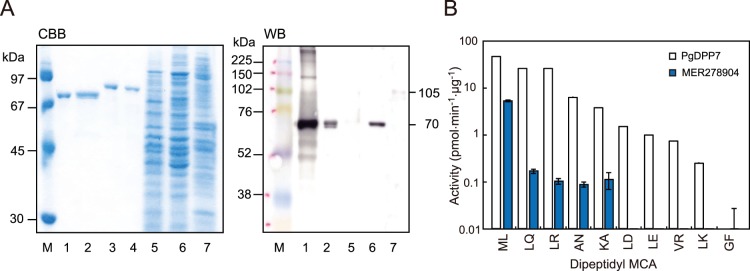
Biochemical analysis of putative PeDPP7 (MER278904). (A) Recombinant PgDPP7 (lane 1), putative PeDPP7 (Leu^3^-Lys^710^, lane 2), PgDPP11 (lane 3), and PeDPP11 (lane 4) (0.3 µg), and aliquots (5 µL) of cell suspensions of *P. gingivalis* ATCC 33277 (lane 5), KDP136 (lane 6), and *P. endodontalis* ATCC 35406 (lane 7) were separated on SDS-PAGE gels, then stained with CBB or subjected to immunoblotting (WB) against anti-PgDPP7 antiserum (300-fold dilution). Peroxidase conjugated anti-rabbit IgG (3,000-fold dilution) was used as the second antibody. Lane M, molecular-weight markers. (B) The hydrolyzing activities (mean±S.D., n = 3) of putative PeDPP7 (MER278904) and PgDPP7 were determined using dipeptidyl MCA.

A C-terminal truncated form (Met^1^-Lys^710^) equivalent to the full-length form of PgDPP7 was successfully expressed, whereas the full-length form was not expressed due to an unknown reason. PeDPP7 Met^1^-Lys^710^ was yielded as a single band of 70 kDa (Fig. 6). Moreover, in accord with the calculated molecular mass of the full length of PeDPP7 Met^1^-Lys^818^ (*Mr = *91,084), a 105-kDa large molecular mass species was detected in whole cell lysates. Under identical conditions, native PgDPP7 was scarcely detected in ATCC 33277, while a 70-kDa band was observed in KDP136 but not in ATCC 33277. PeDPP7 (MER278904) most efficiently cleaved Met-Leu-MCA (Fig. 6) and slowly hydrolyzed Leu-Gln-, Lys-Ala-, Leu-Arg-, and Ala-Asn-MCA. Therefore, the overall specificity mimicked that of PgDPP7, though the activity was substantially lower than that of PgDPP7. Our results showed that MER278904 represents PeDPP7.

## Discussion

The present findings demonstrated that the unassigned *P. endodontalis* S9-family member MER236725 is DPP5 and another unassigned S46-family member, MER278904, represents DPP7. Hence, *P. endodontalis* as well as *P. gingivalis* expresses four DPP molecules, i.e., DPP4 (MER192286), DPP5 (MER236725), DPP7 (MER278904), and DPP11 (MER290751, AB610284). The four DPP activities, especially DPP5 and DPP11, were remarkably increased in *P. endodontalis* cells as compared to the eight *P. gingivalis* strains investigated.

Rgp and Kgp activities were varied among the *P. gingivalis* strains, while they exhibited less variable DPP activities, indicating that there was no strong correlation between DPP and gingipain activities in *P. gingivalis*. On the other hand, the predominant DPP activities in *P. endodontalis* seemed to compensate for the gingipain activity deficit. The dominant hydrolysis of Leu-Asp-MCA mediated by DPP11 was reasonably explained in a previous study by the 4-fold higher *k*_cat_/*K*_m_ value for *P. endodontalis* DPP11 as compared to that of the *P. gingivalis* entity [21].

We previously reported that among the various dipeptidyl-MCAs tested, Lys-Ala-MCA was most predominantly hydrolyzed in *P. endodontalis* and that the activity was significantly higher than that of *P. gingivalis* ATCC 33277 [9], [21]. The present findings clearly demonstrated that the Lys-Ala-MCA-hydrolyzing activity of *P. endodontalis* is predominantly mediated by DPP5. PeDPP5 consists of 691 amino acids with a preference for Ala and hydrophobic amino acids at the P1 position. PeDPP5 is the second entity with expression shown in prokaryotes. Since the PeDPP5 activity was prominent, this enzyme may be involved in its pathogenesis, as also suggested in a study of *Aspergillus* DPP5 [22]. The elevation of Lys-Ala-MCA hydrolysis in *P. endodontalis* was mainly attributed to the 19-fold difference in *k_cat_*/*K_m_* values as the intrinsic enzymatic properties of the two DPP5 molecules. Multiple-fold increases in PeDPP5 gene expression seemed to additionally contribute to the marked elevation. Since an orthologue is not apparently present in humans, DPP5 molecules in pathogenic microorganisms may become a target for antimicrobial agents. Thus, a study of the 3D-structure would be of interest to elucidate the reaction mechanism and also develop antibiotics.

Cell-associated PeDPP7 was detected as an eccentrically large form at 105 kDa and thus suspected to consist of around 818 amino acids, composed of the N-terminal domain and the C-terminal 109-amino acid sequence being rich in Lys (28 residues) and Cys (13 residues). The C-terminal sequence shows no similarity with any others, and seems to be dispensable for the proteolytic activity, since recombinant PeDPP7 (Met^1^-Lys^710^) exhibited the activity and the appropriate substrate specificity. This part might be accidentally tagged as a result of a T-to-G mutation from the stop codon (TGA) into Gly^711^ (GGA) in the PeDPP7 gene. Similarly, a glutamic acid-specific V8 protease, GluV8, belonging to the S46 family was found to be tagged with the C-terminal 48 amino acid residues, which have been shown to be non-essential for the activity [35], [36]. The characteristic GluV8 tag is 12 repeats of the triplet Pro-Asp/Asn-Asn [37]. With the characteristics of the abundant amino acid residues in mind, these C-terminal sequences might be related to the stability and solubility of the peptidases.

An infected root canal has a heterogeneous etiology, with greater than 3 bacterial species generally detected [38]–[43]. A number of studies have reported the prevalence of *P. endodontalis*, as the bacterium was found in 40 of 80 (50%) primary endodontic infection sites [44], 14 of 50 (28%) roots with untreated necrotic pulp [41], and 4 of 24 (18%) specimens obtained from cases of preoperative pain and/or swelling, along with *Fusobacterium nucleatum* (4/4), *Peptostreptococcus micros* (3/4), *Bacteroides forsythus*, *Treponema denticola*, and *Streptpcoccus spp.* (2/4), and *P. gingivalis*, *Prevotella nigrescens*, and *Enterococcus* (1/4) [39]. Recent analysis using pyrosequencing demonstrated that there is a significantly high bacterial diversity (13 phyla, 67 genera) in acute and chronic dental root canal infections, though bacterial richness was not completely revealed in those two types of infections [42]. Therefore, after taking the nutritionally limited condition of the root canal into account, the formation of a bacterial community with other bacterial species may be beneficial for *P. endodontalis* to gain a foothold in this limited niche. Based on results obtained up to this point, the promoted DPP activities seem to mostly contribute to survival of the bacterium.

In conclusion, this study identified and characterized *P. endodontalis* DPP5 and DPP7 as playing key roles together with DPP4 and DPP11 in degradation of extracellular oligopeptides. The four enhanced DPP activities were demonstrated in *P. endodontalis* and, in particular, the remarkable Lys-Ala-MCA-hydrolysis seen was attributed to qualitative and quantitative potentiation of the DPP5 molecule. These findings suggest that high levels of *P. endodontalis* DPP activities compensate for the complete deficit of gingipain-like endopeptidases and enables the bacterium to adapt to the undernourished environment of the root canal.
